# Current Status of Loop-Mediated Isothermal Amplification Technologies for the Detection of Honey Bee Pathogens

**DOI:** 10.3389/fvets.2021.659683

**Published:** 2021-04-12

**Authors:** Timothy C. Cameron, Danielle Wiles, Travis Beddoe

**Affiliations:** ^1^Department of Animal, Plant and Soil Science, Centre for AgriBioscience, La Trobe University, Melbourne, VIC, Australia; ^2^Centre for Livestock Interactions With Pathogens, La Trobe University, Melbourne, VIC, Australia

**Keywords:** honey bee, pathogens, diagnostics, LAMP, in-field, viruses, bacteria

## Abstract

Approximately one-third of the typical human Western diet depends upon pollination for production, and honey bees (*Apis mellifera*) are the primary pollinators of numerous food crops, including fruits, nuts, vegetables, and oilseeds. Regional large scale losses of managed honey bee populations have increased significantly during the last decade. In particular, asymptomatic infection of honey bees with viruses and bacterial pathogens are quite common, and co-pathogenic interaction with other pathogens have led to more severe and frequent colony losses. Other multiple environmental stress factors, including agrochemical exposure, lack of quality forage, and reduced habitat, have all contributed to the considerable negative impact upon bee health. The ability to accurately diagnose diseases early could likely lead to better management and treatment strategies. While many molecular diagnostic tests such as real-time PCR and MALDI-TOF mass spectrometry have been developed to detect honey bee pathogens, they are not field-deployable and thus cannot support local apiary husbandry decision-making for disease control. Here we review the field-deployable technology termed loop-mediated isothermal amplification (LAMP) and its application to diagnose honey bee infections.

## Introduction

The European honey bee, *Apis mellifera*, is a significant component of agricultural systems worldwide. The honey bee is classified as a livestock species due to being a food-producing animal specifically related to honey production (1). Honey bees also produce wax, pollen, royal jelly, and propolis which are all commercial products of the apiary industry. However, honey bees' most significant ecological impact is that they are a critical contributor to food production via pollination (2, 3). The majority of crop species depend on pollination, with some crops such as almonds, onions, sunflowers and avocados being 100% reliant on honey bees for pollination (2, 3). Despite the importance of honey bees to agricultural systems, there have been reports of large scale losses in managed honey bee populations in different parts of the world. These mass losses have been due to various environmental stressors such as pathogens, agrochemical exposure, lack of quality forage, climate change and reduced habitat (4–8).

The prevailing view is that the increasing prevalence of pathogens and parasites are a significant driver in honey bee colony losses. Honey bees are infected by a variety of pathogen and pests such as bacteria (*Paenibacillus larvae, Melissococcus plutonius*) (9), fungi (*Nosema* spp., *Ascosphaerea apis*) (10, 11), mites (*Varroa destructor, Acarapis woodi, Tropilaelaps* spp.) (12) and insect pests such as the greater wax moth (*Galleria melonella*) and the small hive beetle (*Aethina tumidae*) (13). In particular, honey bees are known to be infected by several viruses; most of these are positive-strand RNA viruses belonging to the order of Picornavirales (14). They include several important viruses, such as the Israeli acute bee paralysis virus (IAPV) and the black queen cell virus (BQCV), which belong to the Dicistroviridae family. In contrast, the Iflaviridae family contains the deformed wing virus (DWV) and sacbrood virus (SBV). These viral infections result in deformities, paralysis and/or death; however, most of these viral infections remain asymptomatic until external stress is applied (7, 15). Due to honey bees being predominantly asymptomatic when these virus species are present, molecular-based diagnostic techniques are critical for the accurate diagnosis of infection and making informed management decisions.

## Current Molecular Detection of Honey Bee Pests and Pathogens

The majority of molecular techniques utilize the ability to detect specific pathogen or pest nucleic acids. The most common technique for detecting honey bee pests and pathogens is by quantitative PCR (qPCR), and in the case of a virus, this requires the use of reverse transcriptase to amplify the RNA, which is termed RT-qPCR. There are many individual qPCR assays to detect specific pathogens, such as *P. larvae* (16), *M. plutonis* (17), *A. woodi* (18), *Nosema* spp. (19). Furthermore, a range of viruses [described within (19)] with many of these qPCR tests being multiplexed to perform rapid detection of several pathogens within a single PCR run (20, 21). In recent years, the use of matrix-assisted laser desorption/ionization time-of-flight (MALDI-TOF) mass spectrometry (MS) has emerged as a clinical diagnostic method for the identification of bacterial species (22), and this technology has been applied to honey bee pathogen diagnostics in the identification of different strains of *P. larvae* (23). Despite these techniques' power to identify infected honey bees and hives, they still require specialized labs and trained personnel. The early identification of pests and pathogens in managed honey bees is crucial for the decision-making process regarding disease control, prevention, the strategy of treatment and, therefore, mitigation of the impact of a particular disease. This has been highlighted recently where an integrated management strategy was used to prevent outbreaks and eliminate American foulbrood (*P. larvae*) in a commercial beekeeping operation (24). Several field-based diagnostic technologies that can amplify nucleic acids have emerged in recent years, such as loop-mediated isothermal amplification (LAMP) and recombinase polymerase amplification (25, 26). These technologies have the potential to revolutionize disease management in livestock industries, including honey bees. Currently, the only field-based diagnostic technologies applied for the detection of honey bee pests and pathogens is LAMP. Here we review the current status of field-based nucleic acid amplification techniques, with a particular focus on LAMP, to detect various honey bee pathogens and discuss the implications with which these new diagnostic techniques can impact and inform apiary management practices.

## Principles of LAMP

Loop-mediated isothermal amplification (LAMP) is a novel nucleic acid amplification technology that rapidly amplifies nucleic acids under isothermal conditions (27). LAMP utilizes *Bst or Bsm* DNA polymerase with a strong strand displacing ability and functions at isothermal conditions between 60 and 65°C, thereby eliminating the need for thermal cycling. LAMP does not require an additional reverse transcription step for the amplification of RNA viral gene products and amplifies DNA with high specificity, sensitivity, and speed (27). Additionally, the LAMP amplification is very robust, which is ideal when using a crude DNA extract purified from a range of environmental sources (25). This allows the use of non-invasive sampling techniques to be implemented, such as swabbing hive entrances rather than sampling honey bees themselves, minimizing stress applied to the hive due to excessive human handling. However, to avoid false-positive (due to contamination) and -negative results, in-hive samples/bees should be recommended. These characteristics allow LAMP assays to be performed in field-based settings with cheap and portable equipment (28). It has been proven to be a suitable molecular diagnostic method for field detection of a range of pathogens (29–31).

LAMP reactions are highly specific, involving four primary primers designed to target six distinct sequences on the target DNA (27) (Supplementary Figure 1). These primers are termed the forward and backward inner primer (FIP and BIP) as well as the forward (F3) and backward outer (B3) primer (Supplementary Figure 1). Both the FIP and BIP primers contain complementary regions that bind to form loop structures, providing more sites for primers to bind, initiating further amplification. Optional loop forward (LF) and loop backward (LB) primers can also be added to increase reaction speed by hybridizing to the loop structure to provide additional amplification initiation sites (32). Overall, LAMP is an ideal method for providing cheap (≈AU$ 5–7 per sample), rapid and robust detection of pathogens in-field with high sensitivity and specificity using minimal, simple equipment.

## Application of LAMP for the Detection of Fungal Pathogens of Honey Bees

Nosemosis is a worldwide distributed infectious disease and constitutes a severe problem in both managed European (*A. mellifera*) and Asian honey bee (*A. cerana*) populations as well as wild bumble bees (11, 33, 34). There are three causative agents of nosemosis; *Nosema ceranae* and *N. apis*, which infect both *A. mellifera* and *A. cerana*, while *N. bombi* is a major pathogen of wild bumble bees (34). *Nosema* spp. belongs to a spore-forming fungal family termed microsporidia. They are obligatory unicellular parasites that infect a range of agricultural important livestock (35, 36). In particular, *N. ceranae* is the major pathogen of *A. mellifera* that results in a range of symptoms such as suppressed immune function, lipid synthesis, pheromone and hormone production (37–39). If an infection is severe, it can lead to death and the resulting colonies' collapse (40, 41). Transmission of *N. ceranae* between hives can occur via honey, pollen, nectar and bee fecal matter. It can be controlled by treatment with fumagillin if the infection has been diagnosed in the field. The application of antibiotics has several problems, such as the potential to lead to resistant strains and honey's residue contamination. In many parts of the world, such as the European Union, antibiotics for Nosemosis treatment are banned. There have been three separate LAMP assays developed to detect *N. ceranae* and one for the detection of *N. apis* (Table 1) (42–44). Also, one has been developed for *N. bombi* infection within wild bumble bee populations (45). All three of the *N. ceranae* LAMP assays are extremely sensitive for the detection of infection; however, the LAMP assay developed by Lannutti et al. (44) uses primers targeting the polar tube protein 3 (PTP3) gene, a highly specific and conserved gene of *N. ceranae*. Targeting the *PTP3* gene overcomes non-specific amplification that can occur due to polymorphisms in the 16S rRNA gene, which is the other two assays' target gene (55). Furthermore, only the PTP3-LAMP assay has been successfully evaluated using a panel of field samples (44).

**Table 1:** LAMP assays developed for the detection of pathogens and pests of honey bees.

**Pathogen**	**Target gene**	**Specimen**	**Detection limit**	**Loop primers**	**Field samples^a^**	**References**
**Fungal**						
*Nosema ceranae*	16S rRNA	Adult bees^a^	0.3 ng	No	Yes	(42)
	16S rRNA	Adult bee^c^	100 fg	Yes	No	(43)
	Polar tube protein 3 (PTP3)	Adult bees^b^, Spores	1 pg	Yes	Yes	(44)
*N. apis*	16S rRNA	Adult bee^c^	100 fg	Yes	Yes	(43)
*N. bombi*	SSU rRNA	Adult bees^b^	4.57 × 10^1^ spores/μl	Yes	No	(45)
*Aspergillus flavus*	18S rRNA	Adult bees^b^	N.D.^4^	No	Yes	(46)
	18S rRNA	Adult bees^b^	10^5^ copies/reaction	No	Yes	(47)
**Viral**						
*Sacbrood virus*	SBV-pol gene	Adult bees^b^	10 copies/reaction	Yes	No	(48)
	SBV-pol gene	*cerana indica* Larvae, Pre pupae	N.D	Yes	Yes	(49)
Korean *Sacbrood virus*	VP1 gene	Adult bees^b^, Larvae	1,000 copies/reaction	No	Yes	(50)
Chinese *Sacbrood virus*	VP1 gene	Larvae	1 pg	No	Yes	(51)
**Bacterial**						
*Melissococcus plutonius*	DNA gyrase subunit B	Adult bee^c^, Larvae	2 fg	No	No	(52)
**Insect pests**						
*Aethina tumida* (Small hive beetle)	28S rRNA	*tumida* Adults, Pupae, Eggs	12 pg	yes	No	(53)
*Vespa velutina nigrithorax* (yellow-legged Asian hornet)	COX1 (cytochrome oxidase subunit 1)	*V. velutina nigrithorax* Adults, l Larva, Egg, Nest material	5 pg	yes	Yes	(54)

a *Samples collected from the field*.

b *Pool of adult honey bees were used for extraction of nucleic acid for LAMP assay*.

c *Individual adult honey bee was used for extraction of nucleic acid for LAMP assay*.

d *N.D., not determined*.

Two brood diseases, stonebrood and chalkbrood, are caused by the fungal species *Aspergillus flavus* and *Ascophaera apis*, respectively. Diagnosis based on visual symptoms is difficult as both diseases have very similar clinical symptoms (56). Two LAMP assays have been developed to amplify different regions of the 18S rRNA gene to allow detection of an infected hive (46, 47). In laboratory testing, both assays were shown to be highly specific for the target species only, detecting *A. flavus* with no amplification of *A. apis* and *N. ceranae* DNA. To date, neither test has been optimized for field sampling and detection, with in-field validation of these tests required.

## Application of LAMP for the Detection of Viral Pathogens of Honey Bees

Honey bees can be infected with a range of RNA viruses. However, most of the time, the bees are asymptomatic until external stress is applied, which results in severe loss of honey bees and reduced hive functionality (57). For example, the deformed wing virus (DWV) is often present in low levels in *A. mellifera* with no impacts on hive health; however, the *Varroa destructor* mite's introduction causes the virus to become more virulent, which results in massive mortality (58, 59). Current detection for honey bee viruses has mainly focused on molecular techniques in specialized laboratories (19, 20). Currently, LAMP tests have been developed only for the sacbrood virus (SBV) for country-specific outbreaks (Table 1). SBV causes the larvae to die shortly after capping; however, the disease incidence is higher after stress events such as a shortage of nectar or pollen (60). The assays either amplify the SBV polymerase gene or viral protein gene with a range of sensitivities from 1 pg to 1,000 copies per reaction (Table 1). The range of sensitivity of detection is most likely due to sampling processing. Apart from SBV, there is significant scope for developing LAMP assays to detect a range of viruses that affect honey bees, particularly DWV. Currently, Australia is free of DWV (as well as the Varroa mite), thus has not suffered large colony loss due to these pathogens. It would be advantageous to have an assay to detect this virus in honey bees imported into the country to maintain Australia's DWV free status (61).

## Application of LAMP for the Detection of Bacterial Pathogens of Honey Bees

Of the several pathogenic bacteria species that cause disease of managed honey bees, there are only two economically relevant pathogens, *Paenibacillus larvae* and *Melissococcus plutonius*, the causative agents of American Foulbrood (AFB) and European Foulbrood (EFB), respectively (9). *P. larvae* and *M. plutonius* are listed by OIE (World Organisation for Animal Health) as category B organisms, which are defined as disease-causing agents considered to be of socio-economic and/or public health importance within countries. There are limited control options against theses pathogens; antibiotics such as Terramycin are effective against *M. plutonius* though in many parts of the world such European Union antibiotic treatments are strictly prohibited while there are no effective treatment options for *P. larvae* as antibiotics will not kill the infective spore stage thus burning of infected hives are required to minimize the spread of the pathogen (62). Prevention is better than the cure, and to this aim, there are commercial lateral flow devices that detect AFB and EFB infections; however, they all work only on larval samples. However, there is a demand for highly sensitive field tests from environmental samples to aid in disease control, prevention, monitoring and treatment strategies for bacterial diseases of honey bees. The predominant method utilized to distinguish the two diseases is visual inspection, which requires subjective expertise. Given the differences in treatment of AFB and EFB, more precise diagnostic methods are urgently required for use in the field. Currently, there is only a LAMP test for *M. plutonius*, which is extremely sensitive (detection limit of 2 fg) on laboratory prepared samples; however, this test has not been used in the field. Future work should be directed toward developing and validating LAMP assays for the causative agents of AFB and EFB in the field. A multiplex assay for the differentiation of these two species would be ideal.

## Application of LAMP for the Detection of Insect Pests and Mites of Honey Bees

Several species of insect pests and mites of *A. mellifera* can cause significant problems in commercial apiaries worldwide. The *Varroa destructor* mite is the primary biotic cause of colony collapse syndrome and is found in nearly every continent except Australia (63, 64). Other exotic pests such as the small hive beetle (SHB, *Aethina tumida*) and various species of hornets that have been introduced into a range of non-native countries can have a devastating effect on honey bees (65, 66). The ability to rapidly and reliably identify invasive species at all life stages, and within the nest and hive debris is crucial in mounting an effective control response. As LAMP technology is well suited to aid biosecurity, there have been two reported LAMP assays for insect pests of honey bees (Table 1). The small hive beetle LAMP assay targets the 28S ribosomal gene and is able to detect the presence of SHB DNA down to 12 pg within 20 min (53). True hornets belong to the *Vespa* family and are naturally found only in Asia, Europe and Africa. They all prey on other insects, including honey bees; thus, introductions into non-native areas can have severe consequences for honey bee populations in these areas. Only one of the 20 species of true hornets have had a LAMP assay developed for their identification (Table 1). The yellow-legged Asian hornet (*Vespa velutina nigrithorax)* has been rapidly spreading throughout Europe after being accidentally introduced into France from China (67). The LAMP assay can reliably identify all life stages of *V. v. nigrithorax* as well as from nest material as low as 5 pg in 10 min. Both assays allow rapid unequivocal identification of insect pests which are normally identified via manual inspection of morphological features. An additional benefit of using LAMP is it can provide identification on decomposing or incomplete insect samples; thus, these assays will be useful in control programs to limit the damage caused by these pests. In the future, an entire suite of LAMP assays should be developed for the rapid identification of insect pests of honey bees to aid effective biosecurity control measures.

## Application of LAMP for In-field Detection of Pests and Pathogens of Honey Bees

Several LAMP assays have been developed to detect pests and pathogens of honey bees; however, none have been applied in the field. The current sampling methods for detecting pathogens require the use of specialized equipment in a laboratory setting. Further research is required to establish methods for lysing honey bees in the field such as the use of ball bearing and small capped tubes which have been used previously for plant and insect samples (68). The majority of LAMP assays are performed in an 8-strip tube, thus providing a negative and positive control and six tubes for testing. The six sample tubes could contain a single or duplex reaction and have the ability to analyze six different samples or you can have six different individual assays and the ability to use a single sample. What configuration the field-based LAMP test kits are will be determined mainly by consumer demand.

## Conclusions

The increasing awareness about the important roles honey bees play in food production and security has led to many advances in understanding honey bees' health and well-being. Honey bees are under threat from a range of environmental stress and infection from a large variety of pathogens. The ability to identify specifically and rapidly infection at the hive-site will allow for improved management and treatment strategies. LAMP assays in the last few years have become an important tool to aid in the detection of both exotic and endemic pathogens in the livestock industry. Several LAMP assays have been developed for honey bee pathogens, with a number of these still requiring in-field validation to confirm its use as an on-site diagnostic tool. It is important that researchers continue to develop assays against other honey bee pathogen and promote them for use in the field, with consideration given to non-invasive sampling methods to maximize the benefit from LAMP assays and reduce stress on honey bee hives introduced by humans.

## Author Contributions

TC, DW, and TB conceived, designed, and wrote the manuscript. All authors contributed to the article and approved the submitted version.

## Conflict of Interest

The authors declare that the research was conducted in the absence of any commercial or financial relationships that could be construed as a potential conflict of interest.
