# Supplementary figures and images for: Current Status of Loop-Mediated Isothermal Amplification Technologies for the Detection of Honey Bee Pathogens
Source: Front Vet Sci. 2021 Apr 12;8:659683. doi: 10.3389/fvets.2021.659683 (PMC8071855; doi:10.3389/fvets.2021.659683)

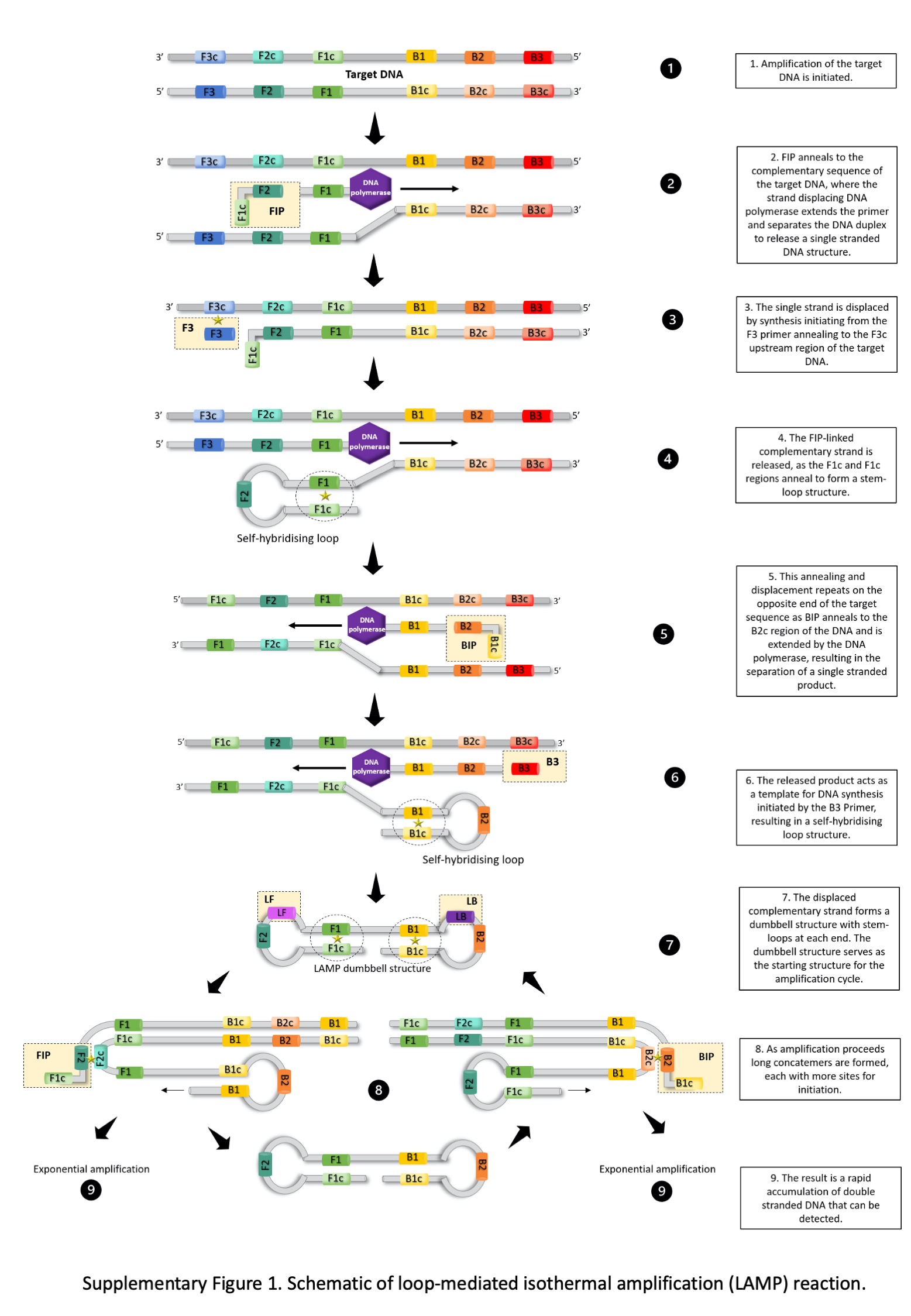

Supplement: Supplementary file 1 [file Figure_1.JPEG]
